# To Our Readers

**Published:** 1860-06

**Authors:** 


### EDITORIAL DEPARTMENT.

— The large amount of original matter, and the presence of an index in this number, must be our apology for the brevity of our editorial. Several reviews and notices of books have been deferred to another month, and the Proceedings of Societies we have been obliged to omit altogether. In commencing a new volume, we shall endeavor to make such a disposition of our pages that each department shall receive its proper quota, and a greater variety be given to our readers. We have to beg the indulgence of those of our correspondents whose articles have been deferred, and in closing this volume, we do so with the intention of improving upon it in our next.

